# Insights Into Pediatric Asthma: A Population Study From Alto Minho Health Center

**DOI:** 10.7759/cureus.52577

**Published:** 2024-01-19

**Authors:** Catarina Soares, Daniela Alves, Soraia Gonçalves, Tomás Martins, Patricia Coelho, Virginia Laranjeira, Joana Pedrosa, Daniela Lisboa, Marco Fernandes, Mariana Branco

**Affiliations:** 1 Family Medicine, Unidade Local de Saúde do Alto Minho, Viana do Castelo, PRT; 2 Psychology, Life and Health Sciences Research Institute (ICVS) School of Medicine, University of Minho, Braga, PRT; 3 Pediatrics, Unidade Local de Saúde do Alto Minho, Porto, PRT

**Keywords:** food allergy, atopic dermatitis, allergic rhinitis, skin prick tests, exacerbation factors, exposure factors, pediatric asthma

## Abstract

Background

Asthma represents one of the most common diseases in childhood, with a prevalence ranging between 9% and 13% in Portugal. Therefore, it holds significant importance in pediatric health. While existing studies have shed light on asthma in the Portuguese population, they have predominantly concentrated on urban centers, with the population of Alto Minho remaining underrepresented in the literature. This study aims to understand the main factors of exposure, exacerbation, and the most prevalent allergens in a pediatric sample from the Alto Minho Local Health Unit, Portugal.

Methodology

A retrospective cohort study was conducted among 239 pediatric asthma patients aged between five and 18 years at the Alto Minho Health Center. Data on demographics, clinical information, family history, environmental exposures, exacerbating factors, and prick test results were analyzed.

Results

Of the 239 patients, 64.44% were male and 35.56% were female. The majority of the sample exhibited a normal body mass index (82.17%) and a family history of atopy (66.67%). Noteworthy patterns emerged in comorbidities, notably an increased association with allergic rhinitis, the most frequent concomitant atopic pathology (79.50%), followed by atopic dermatitis (27.61%) and food allergy (10.88%). Sensitization to dust mites, particularly *Dermatophagoides pteronyssinus*, was widespread among the participants. Environmental exposures were marked by significant factors such as proximity to plants and trees, soft toys, and living in rural areas. Exacerbating factors included common triggers such as exercise, seasonal variations, and even laughter. Statistically significant associations were found between atopic comorbidities, exacerbation factors, exposure factors, and prick test results.

Conclusions

Our findings align with global trends, emphasizing the prevalence of atopic pathologies in pediatric asthma. Sensitization patterns and environmental exposures are indicative of regional influences. Study limitations include sample size and data standardization issues. Despite these limitations, the study significantly contributes to understanding pediatric asthma in Alto Minho, offering valuable insights for prompt diagnosis and targeted treatments.

## Introduction

Asthma is a complex and heterogeneous disease, characterized by chronic airway inflammation. It is characterized by respiratory symptoms, including wheezing, dyspnea, chest tightness, and cough, which can vary in frequency and severity and can be associated with variable expiratory airflow limitation [1].

Asthma is a significant health problem worldwide and is one of the most common chronic diseases in childhood, with prevalence rates ranging from 1% to 18% across different countries [2]. In Portugal, data from the International Study of Asthma and Allergies in Childhood revealed that the prevalence of asthma in children aged six to seven years was 13%, while in teenagers aged 13 to 14 years, it ranged from 9% in 1995 to 12% in 2002 [3].

Numerous studies have demonstrated a notable association between childhood asthma and atopic diseases, including allergic rhinitis (AR), atopic dermatitis (AD), and food allergies (FAs) [3,4]. It is known that over 80% of asthmatics have comorbid AR [5]. Asthma, AR, and AD are known as the *atopic triad* and share immunological mechanisms [6].

Several risk factors have been identified, such as a personal history of AR, AD, or FAs; a family background of allergic diseases; and a maternal history of asthma and/or rhinitis [1,3,6,7,8].

The main hypothesis that is currently proposed to explain the onset of allergic diseases is an epithelial barrier defect. Thus, the atopic march could correspond to epithelial dysfunction, self-sustained by a secondary allergenic sensitization, explaining the transition from AD to allergic asthma. Furthermore, AD severity appears to be a risk factor for associated FAs. Results from population-based, birth, and patient cohorts have shown that early-onset and severe AD, male gender, parental history of asthma, and early and multiple sensitizations are risk factors leading to atopic march and the development of asthma [9].

A Portuguese study revealed that in individuals who experience both AR and asthma, the most prevalent allergens are pollens (wild and cultivated grasses) along with dust mites (*Dermatophagoides pteronyssinus*, *Lepidoglyphus destructor*, and *Dermatophagoides farinae*) [10].

As delineated in the literature, FA is a firmly established risk factor in the onset of allergic asthma among children. Studies have indicated that children with FAs are potentially twice as likely to develop asthma [1,7].

Triggers of asthma exacerbations mostly include airborne allergens, respiratory infections, and occupational substances. Other factors that can influence asthma include tobacco smoke, gastroesophageal reflux disease, environmental pollutants, some medications such as β-blockers and non-steroidal anti-inflammatory drugs, and food additives. Irritants such as exercise in cold air, emotions, stress, strong odors or other respiratory irritants, and temperature changes can trigger bronchoconstriction. Animal dander and house dust mites have been shown not only to contribute to the development of asthma but also to induce exacerbations [11].

The burden of asthma on pediatric health in Portugal deserves considerable attention due to its significant prevalence. However, a knowledge gap exists regarding the rural context, with most studies focusing on urban centers. Thereby, the population of Alto Minho remains underrepresented in the literature, warranting a comprehensive understanding to facilitate tailored medical interventions for preventive measures, diagnoses, and ongoing care among the pediatric demographic of this region.

The main aim of this study is to comprehensively characterize a sample of children and adolescents diagnosed with asthma under the care of specialized pediatric practitioners in Alto Minho. Through this effort, our aim is twofold: first, to enhance the understanding of potential exposure and exacerbating factors specific to this population, thus bridging a crucial gap in the existing literature. Second, we seek to identify the most prevalent allergens within this sample, enriching our insights into this demographic.

## Materials and methods

Ethical considerations

This study was conducted following the Declaration of Helsinki (59th Amendment) and was approved by the relevant local ethics review board from the Health Center of Alto Minho, Portugal (approval number: 37/2023). The anonymity and confidentiality of the study participants were guaranteed throughout all research activities.

Characterization of participants

A retrospective cohort study was conducted among patients who were under follow-up on December 31, 2020, in the pediatric unit of Unidade Local de Saúde do Alto Minho, located in north Portugal. This study included information from patients aged between five (the ability to diagnose asthma in preschool children is controversial) and 18 years with a confirmed diagnosis of asthma [12]. Children who did not meet the inclusion criteria were excluded.

**Figure 1:**
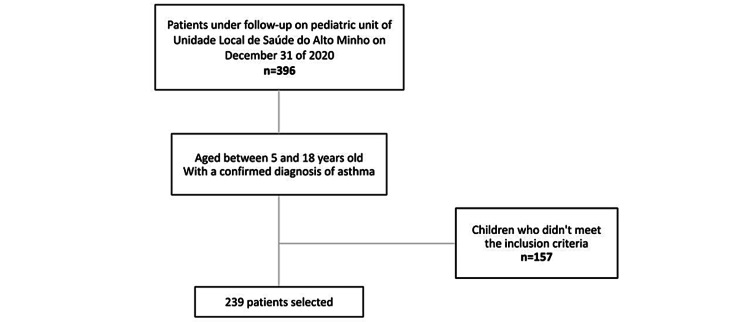
Flowchart showing the selection process.

The data used in this study encompassed the gender and age of children and adolescents, clinical information such as body mass index (BMI) and other atopic diseases (AR, AD, and FA), family history of first and second-degree relatives with atopic diseases, exposure factors (e.g., animals, pollens, smoke, humidity, rugs and curtains, urban or rural residential area), exacerbating factors (e.g., laughter, physical exercise, cold, season, smoke), and skin prick test results. Taking into account that it can be considered a risk factor, BMI was collected from the first appointment registers. Following the established criteria, 239 patients were selected (Figure 1).

Statistical analysis

Descriptive statistics of clinical and demographic data were performed using SPSS Statistics version 27 (IBM Corp., Armonk, NY, United States). Data were presented as frequency and percentage for categorical variables and as mean, standard deviation, median, and minimum/maximum values for numerical variables, both for the total sample and specific subgroups. As the data did not follow a normal distribution, non-parametric tests, such as the chi-square test, were applied. Results were considered statistically significant when the p-value was less than 0.05.

## Results

Demographic and body mass index analysis

**Figure 2:**
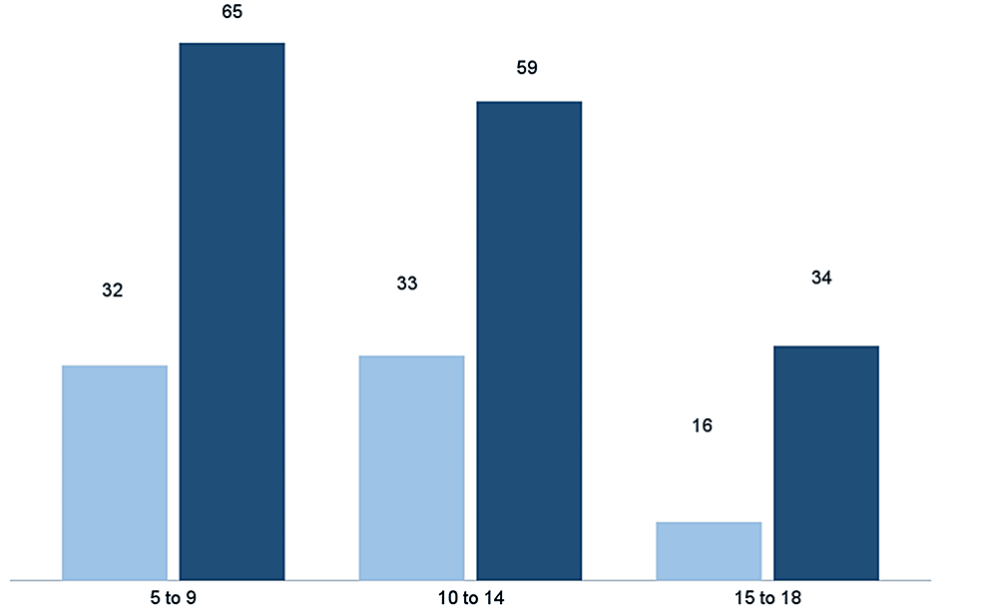
Schematic representation of the participants’ age distribution. The data are represented as N. Sample: n = 239. Dark blue = male individuals; light blue = female individuals

A total of 239 patients diagnosed with asthma were enrolled in the present study. Concerning sex distribution, the sample comprised 154 (64.44%) males, exhibiting a mean age of 13.60 years (SD = 3.99), and 85 (35.56%) females, with a mean age of 13.65 years (SD = 4.13). Figure 2 delineates the age distribution of patients, at the time of data collection, stratified by gender.

**Figure 3:**
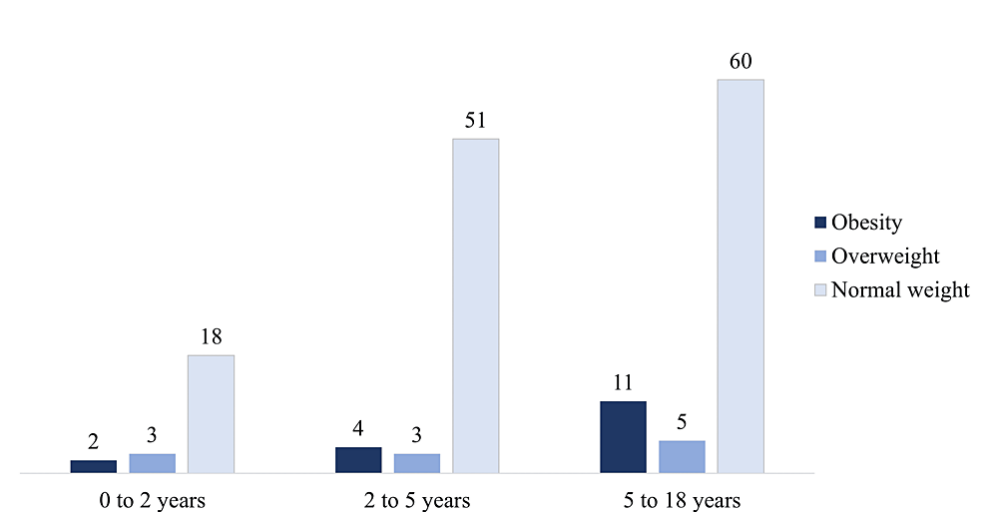
Schematic representation of the total sample according to different body mass index levels. The data are represented as N. Sample: n = 157.

Due to heterogeneity in registration, BMI data were successfully obtained for a subset of 157 participants on the day of the first appointment. Table 1 delineates the descriptive statistics of the BMI measurements of the overall sample, according to the World Health Organization percentile (obesity > P97; overweight > P85). Figure 3 illustrates the distribution of patients within various BMI categories.

**Table 1:** Distribution of the total sample, stratified by age according to the different body mass index (BMI) levels. The data are represented as N and percentage (%). Sample: n = 157.

BMI	0 to 2 years		2 to 5 years		5 to 19 years		Total	
	n = 23	%	n = 58	%	n = 76	%	n = 157	%
Obesity	2	8.70	4	6.90	11	14.47	17	10.83
Overweight	3	13.04	3	5.17	5	6.58	11	7.01
Normal weight	18	78.26	51	87.93	60	78.95	129	82.17

Overall, 17 (10.87%) children/adolescents were obese, and 11 (7.01%) were overweight; however, the majority, 129 (82.17%) children/adolescents were of normal weight. The average percentile of the sample was 68.68 (SD = 27.97) and was located within the range of normal weight. For more details, see Table 1.

Atopic diseases, family history, and contextual factors

Of the 239 children and/or adolescents with asthma, 221 (92.47%) had comorbidity with another atopic condition (i.e., AR, AD, and FA). Here, data are described for those with asthma and only one other condition, those with asthma and two other associated conditions, and, finally, those with AR, AD, and FA combined with a previous diagnosis of asthma.

**Figure 4:**
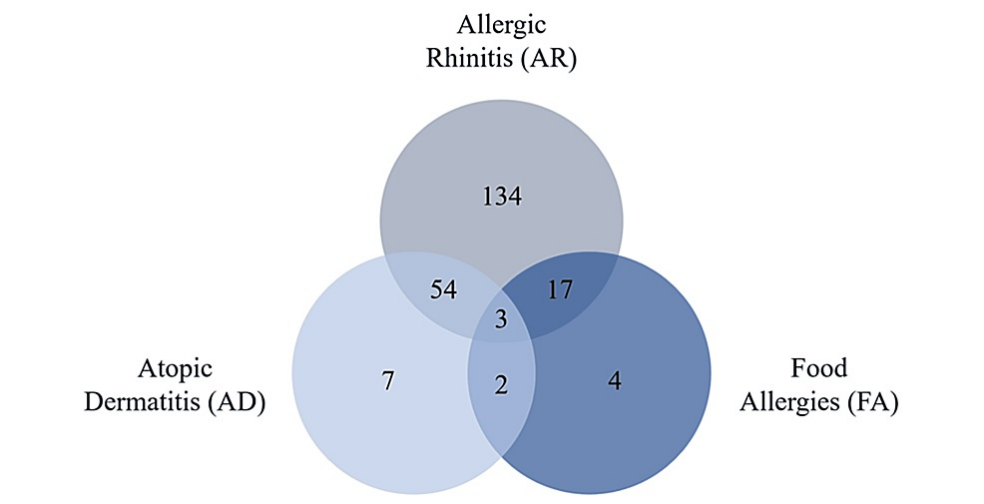
Schematic representation of the total sample according to atopic diseases (allergic rhinitis, atopic dermatitis, and food allergies). The data are represented as N. Sample: n = 221.

Regarding other atopic diseases, 145 (60.67%) of the children and/or adolescents had only one more comorbidity besides asthma: 134 (56.07%) had AR, seven (2.93%) had AD, and four (1.67%) had FA. The remaining individuals presented with combined conditions of two or three atopic diseases, meaning that 54 (22.59%) individuals had both AR and AD, 17 (7.11%) had AR and FA, two (0.84%) had AD and FA, and, finally, three (1.26%) had AR, AD, and FA (Figure 4).

In the present sample (n = 239), 100 (41.84%%) children had a positive family history of atopy; 55 (23.01%) of the individuals had a maternal history, and 47 (19.67%) had a paternal history. Notably, 16 (6.69%) of these children and adolescents had a maternal and paternal history (Table 2).

**Table 2:** Number of children and adolescents with parents diagnosed with atopic diseases. The data are represented as N and percentage (%). Sample: n = 221.

Family history		
Mother		
	n = 55	%
Asthma	20	36.36
Allergic rhinitis	35	63.63
Atopic dermatitis	5	9.09
Food allergies	2	3.63
Father		
	n = 47	%
Asthma	21	44.68
Allergic rhinitis	27	57.45
Atopic dermatitis	4	8.51
Food allergies	0	0

In addition to parental history (n = 221), 17 (7.69%) children and adolescents reported a second-degree family history of atopic diseases. Additionally, 11 (4.98%) individuals had brothers and/or sisters with atopic conditions.

Regarding environmental exposure (n=239), 91 (38.08%) children and/or adolescents reported exposure to pollens; 57 (23.85%) to soft toys, rugs, and curtains; 51 (21.34%) to pets; 27 (11.30%) to passive smoking; and 20 (8.37%) to humidity. Additionally, 65 (27.20%) lived in urban areas, and 158 (72.80%) lived in rural areas.

Considering the exacerbation factors (n = 239), 67 (28.03%) individuals reported exercise, 45 (18.83%) seasonal exacerbation, 26 (10.88%) laughter, four (1.67%) cold weather, and none reported smoke.

**Figure 5:**
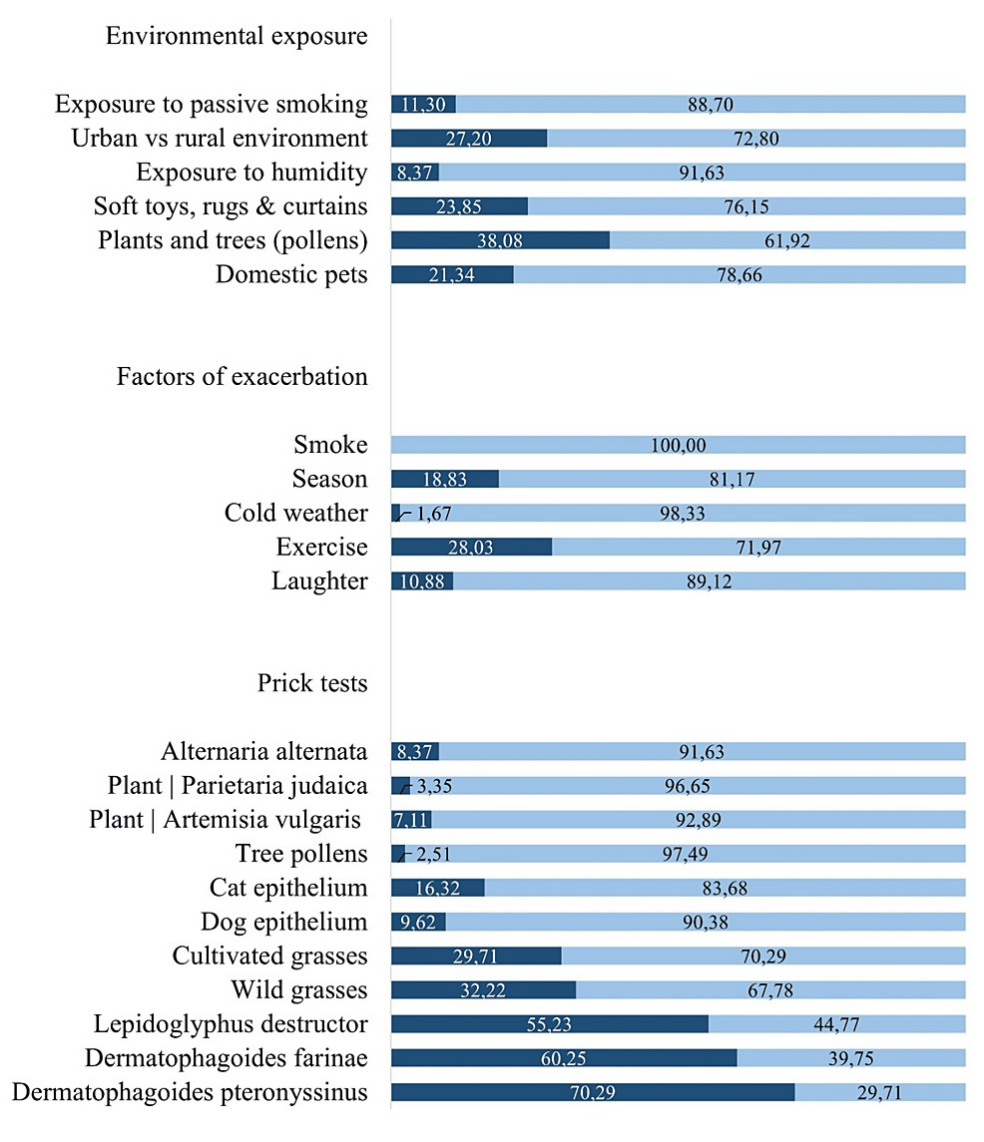
Schematic representation of environmental exposures, exacerbation factors, and skin prick test results. The data are represented as percentage (%). For the majority of variables: dark blue = yes; light blue = no. Considering urban vs. rural environment: dark blue = urban; light blue = rural

Of the 239 children and/or adolescents with asthma, 56 did not have available prick test results. Of the sample (n = 183), 168 (70.29%) tested positive for *Dermatophagoides pteronyssinus*, 144 (60.25%) for *Dermatophagoides farinae*, and 132 (55.23%) for *Lepidoglyphus *destructor. After dust mites, wild grasses and cultivated grasses followed, with 77 (32.22%) and 71 (29.71%) positive results, respectively (Figure 5).

Moreover, chi-square tests were conducted to examine the relationship between atopic diseases (i.e., AR, AD, and FA) and family history as well as contextual factors. Indeed, AR was found to be associated with exposure to pollens (χ^2^ (1) = 2.04, p = 0.012) and prick test results positive to *Dermatophagoides pteronyssinus* (χ^2^ (1) = 30.42, p < 0.001), to *Dermatophagoides farinae* (χ^2^ (1) = 19.22, p < 0.001), to *Lepidoglyphus *destructor (χ^2^ (1) = 9.01, p =0.003), and to wild grasses (χ^2^ (1) = 4.72, p < 0.030).

AD was found to be associated with AR in second-degree relatives (χ^2^ (1) = 5.17, p = 0.023), exposure to urban environments (χ^2^ (1) = 3.93, p = 0.048), and with the following exacerbating factors: the practice of physical exercise (χ^2^ (2) = 6.47, p = 0.039), the season of the year (χ^2^ (2) = 6.91, p = 0.045), and smoke (χ^2^ (1) = 4.82, p = 0.028). Additionally, the prick test results considering certain pollens and fungi showed an association, specifically *Artemisia vulgaris* (χ^2^ (1) = 6.40, p = 0.011) and *Alternaria alternata* (χ^2^ (1) = 4.02, p = 0.045).

Finally, FA was associated with AD (χ^2^ (1) = 5.58, p = 0.018) and FA manifested by maternal history (χ^2^ (1) = 3.86, p = 0.050).

## Discussion

This study aimed to characterize a pediatric sample of 239 pediatric patients diagnosed with asthma in Alto Minho. Gender distribution matched with other international studies, which reported a predominance of allergic pathology in males in the pediatric age group [13].

BMI might be one of the potential risk factors for developing asthma, but the evidence is controversial [5]. Although some studies have shown a significant relationship between BMI (obesity/overweight) and asthma among children and adolescents, according to Inquérito Nacional de Controlo de Asma [14], the Portuguese national survey of asthma control, poorer symptom control was observed in the overweight and obese groups, but the majority of patients had a normal BMI. Our results showed that the majority of the sample was normal weight, which aligns with the results of the national study. Additionally, no significant age or BMI differences were found between genders. However, more robust future studies are needed to draw conclusions.

In line with global research, there is a high prevalence of other atopic pathologies in children/adolescents with asthma in this geographical region [13,15]. Most individuals also had a diagnosis of AR. In addition, the atopic triad was present in 22.60% (n = 54) of the sample studied. Of the children and adolescents reporting atopic diseases, most had a family history of atopy.

The prevalence of sensitization to dust mites was found to be high, followed by pollens, which is consistent with studies indicating that dust mites and pollen allergies are the most common causes of respiratory allergic diseases in Portugal and other European countries [15].

The most prevalent mite in the study was *Dermatophagoides pteronyssinus*, and this prevalence is related to the fact that this species is the most frequent in homes in the northern coastal area of the country, as shown on the acarological map of Portugal [15]. Although the *Dermatophagoides farinae* mite has a low presence in the northern coastal region of Portugal, in this study, it was the second most prevalent mite causing atopic disease. The third most prevalent mite in this study was *Lepidoglyphus *destructor, which aligns with the overall mite prevalence observed in the Portuguese territory.

The main environmental exposure factors were plants and trees, soft toys, rugs and curtains, and domestic pets. Furthermore, most individuals lived in rural areas; however, it is not possible to establish a relationship with asthma in this group because the geographical area corresponding to this sample was mostly rural.

We believe that the high prevalence of sensitization, seen in all allergological pathologies and all age groups, may reflect the influence of these housing and environmental conditions in the northern coastal area of Portugal, which leads to early and daily exposure to these allergens.

The main exacerbating factors recorded were exercise, season, and laughter, all of which have been shown in the literature to cause bronchoconstriction [1]. However, we would like to highlight the lack of data, as there was often no reference to it in the patient record.

Similar to another study from Portugal, the authors corroborated various associations between atopic diseases and contextual factors. For instance, individuals with both asthma and AR were associated with exposure to certain plants and dust mites [10]. In our study, we also found associations between AD and AR in second-degree relatives, urban environments, and the presence of specific plants.

Limitations and future directions

The fact that this study was conducted on a limited size and convenience sample, encompassing patients who consecutively attended the pediatric unit of Unidade Local de Saúde do Alto Minho, are two important limitations of this study. Additionally, the data gathered from pediatric appointment records lacked standardization, leading to a lack of information on some variables. Furthermore, the exclusive focus on individuals with asthma prevents comparisons to the non-asthmatic subset. It would be interesting to conduct further research investigating the contrast between this study’s findings with those of individuals who do not suffer from asthma while highlighting the disparities in risk factors or family history.

Despite some limitations, this study improves the characterization of the pediatric asthmatic population in the Alto Minho region, resulting in an important opportunity to increase the understanding of the disease in younger patients who exhibit risk factors. This newfound insight will hopefully lead to prompt diagnoses and effective treatments.

The characterization of the pediatric population of this area allows pediatricians and family doctors, who serve as primary hubs for those seeking allergic pathology advice, to stay alert regarding allergy signs and provide better care. Testing asthma patients for allergens allows them to be identified and for environmental avoidance measures and targeted therapeutic strategies to be implemented.

## Conclusions

In summary, our study on pediatric asthma in Alto Minho, Portugal, reveals significant associations between asthma and atopic conditions, highlighting local sensitization patterns and environmental influences. While acknowledging limitations, including sample size constraints, this study provides crucial insights for prompt diagnosis and targeted treatment. The findings underscore the importance of considering family history and regional factors in managing pediatric asthma. However, further investigation is required regarding the associations between atopic disease and contextual factors. Authors acknowledge that larger and more diverse studies are warranted to validate and expand upon these findings, but this research lays a foundation for improved care in the pediatric population of Alto Minho.
